# Functional and quality of life outcomes after partial glossectomy: a multi-institutional longitudinal study of the head and neck research network

**DOI:** 10.1186/s40463-017-0234-y

**Published:** 2017-09-04

**Authors:** Agnieszka Dzioba, Daniel Aalto, Georgina Papadopoulos-Nydam, Hadi Seikaly, Jana Rieger, Johan Wolfaardt, Martin Osswald, Jeffrey R. Harris, Daniel A. O’Connell, Cathy Lazarus, Mark Urken, Ilya Likhterov, Raymond L. Chai, Erika Rauscher, Daniel Buchbinder, Devin Okay, Risto-Pekka Happonen, Ilpo Kinnunen, Irjala Heikki, Tero Soukka, Juhani Laine, Head and Neck Research Network

**Affiliations:** 1 Department of Surgery, University of Alberta Hospital, Edmonton, AB T6G 2G4 Canada; 2 Institute for Reconstructive Sciences in Medicine (iRSM), Misericordia Community Hospital, Edmonton, AB Canada; 3 Rehabilitation Medicine, Communication Sciences and Disorders, University of Alberta, Edmonton, AB Canada; 4 Division of Otolaryngology-Head and Neck Surgery, University of Alberta, Edmonton, AB Canada; 5 Division of Head and Neck Surgery, Department of Otolaryngology, Head & Neck Surgery, Mount Sinai Beth Israel, New York, NY USA; 6 Thyroid, Head And Neck Cancer (THANC) Foundation, New York, NY USA; 7 Department of Oral and Maxillofacial Surgery, Mount Sinai Beth Israel, New York, USA; 8 Department of Oral and Maxillofacial Diseases, Turku University Hospital, Turku, Finland; 9 Department of Oral and Maxillofacial Surgery, University of Turku, Turku, Finland; 10 Department of Oto-Rhino-Laryngology, Turku University Hospital, Turku, Finland

**Keywords:** Oral cancer, Quality of life, Speech, Swallowing, Patient-reported outcome

## Abstract

**Background:**

While aggressive treatment for oral cancer may optimize survival, decrements in speech and swallowing function and quality of life often result. This exploratory study investigated how patients recover their communicative function, swallowing ability, and quality of life after primary surgery [with or without adjuvant (chemo)radiation therapy] for tongue cancer over the course of the first year post-operation.

**Methods:**

Patients treated for oral cancer at three institutions (University of Alberta Hospital, Mount Sinai Beth Israel Medical Center, and Turku University Hospital) were administered patient-reported outcomes assessing speech [Speech Handicap Index (SHI)], swallowing [(M.D. Anderson Dysphagia Inventory (MDADI)] and quality of life [European Organization for Research and Treatment of Cancer Quality of Life Questionnaire Head and Neck Module (EORTC-H&N35)]. Outcome measures were completed pre-operatively and at 1-, 6-, and 12-months post-operatively.

**Results:**

One hundred and seventeen patients undergoing partial glossectomy with reconstruction participated in this study. Results indicated no significant differences in swallowing function (MDADI and EORTC-H&N35 subscales) between baseline and 6 months post-surgery and no significant differences in speech function (SHI subscales) between baseline and 1 year post-surgery. Most quality of life domains (EORTC-H&N35 subscales) returned to baseline levels by 1 year post-operation, while difficulties with dry mouth and sticky saliva persisted. A clear time trend of adjuvant (chemo)radiation therapy negatively affecting dry mouth scores over time was identified in this study, while negative independent effects of chemoradiation on MDADI swallowing, and EORTC-H&N35 swallowing, eating, and opening mouth subscales were found.

**Conclusions:**

Assessment time influenced patient-reported speech, swallowing, and quality of life outcomes, while treatment (by time) effects were found for only swallowing and quality of life outcomes. Results of the present study will help guide clinical care and will be useful for patient counseling on expected short and long-term functional and quality of life outcomes of surgical and adjuvant treatment for oral cavity cancer.

**Electronic supplementary material:**

The online version of this article (10.1186/s40463-017-0234-y) contains supplementary material, which is available to authorized users.

## Background

Individuals suffering with cancer of the oral tongue undergo multiple treatment regimens. The standard of care for patients presenting with a tumour in the anterior tongue includes partial glossectomy, as it offers the best survival benefit to the patient [1–4]. Furthermore, adjuvant radiation therapy (RT) or chemoradiation therapy (CRT) are also often indicated [5–7]. These aggressive combinations of treatment modalities have resulted in significant improvement in survival outcomes, but the effects on quality of life and survivorship remain profound [5, 8, 9].

Recently, patient and clinician driven interest in functional outcomes related to different treatment regimens have highlighted the need for this information to customize treatment for individual patients while optimizing survival and function. Current literature assessing functional outcomes has lacked standardization in the tools used, follow-up times and reporting methods, making it difficult to draw conclusions in a systematic way [9–12].

The Head and Neck Research Network (HNRN) is a multi-disciplinary international research organization dedicated to advancing treatment for head and neck cancer (HNC) through functional outcomes research. This network was founded by three partnering international centers in Edmonton, Alberta, Canada; Turku, Finland; and, New York, New York, USA. The HNRN maintains an international database with clinically-significant data and integrity, allowing for the collection of outcomes data in a standardized fashion and facilitating the conduct of collaborative research on function and quality of life in patients with HNC.

Individuals suffering with oral cancer experience life altering deficits, due in most part to treatment effects on the tongue, jaws, throat, salivary glands, and the sensory systems of the head and neck [10, 13–15]. This in turn results in severe deficits in speaking, eating, and appearance, significantly impacting the physical and psychosocial health of patients [10, 11, 15, 16]. Furthermore, it is well acknowledged that treatment modality may differentially affect the functioning and quality of life of patients with oral cavity cancer [5, 6, 9].

The purpose of the present exploratory study of the HNRN was to answer the following research questions:

1) Are there differences in self-perceived functional and quality of life outcomes between pre-treatment and at three post-operative time-points (1-, 6-, and 12-months) in patients treated with primary surgery (with or without C/RT) and reconstruction for cancer of the oral tongue?

2) Are speech, swallowing, and quality of life outcomes influenced by treatment modality [primary surgery only (Sx), primary surgery and adjuvant radiation therapy (Sx-RT), primary surgery and adjuvant chemoradiation therapy (Sx-CRT)] in patients with oral cancer?

## Methods

This multi-institutional longitudinal study was conducted at three HNRN centers between January 2010 and September 2015. The centers included: the University of Alberta Hospital and Institute for Reconstructive Sciences in Medicine in Edmonton, Alberta, Canada; Turku University Hospital in Turku, Finland; and Mount Sinai Beth Israel Medical Center in New York, New York, USA. Prior to commencement of data collection, this study received ethical approval from the Health Research Ethics Boards of each participating institution.

### Participants

Patients undergoing primary surgery for cancer of the oral cavity (anterior two-thirds of the tongue) were prospectively recruited. All patients were diagnosed with SCCA and were treated with a partial glossectomy with or without floor of mouth resection and with or without resection of the mandible. Reconstruction was completed with a free tissue transfer (most commonly radial forearm free flap) when deemed appropriate by the resection surgeon. When indicated, patients also received adjuvant C/RT for their cancer treatment. Dose of radiation ranged between 60 to 72 Gy. Patients for whom the resection had extended to other structures of the oral and oropharyngeal areas (e.g., buccal mucosa, base of tongue, etc.), patients who received previous oncological treatment in the head and neck region, and patients with existing speech or swallowing disorders or cognitive impairment were excluded from the study.

### Outcome measures

Patients attended pre-treatment and post-surgical appointments at their respective medical center to assess their functional status related to speech and swallowing, as well as quality of life. As part of a standard clinical protocol, the following patient-reported outcomes were administered to and self-completed by patients in a quiet, private setting: 1) the Speech Handicap Index (SHI) [12, 17, 18]; 2) the M.D. Anderson Dysphagia Inventory (MDADI) [19–21]; and, 3) the European Organization for Research and Treatment of Cancer Quality of Life Questionnaire Head and Neck Module (EORTC-H&N35) [22–24] (see Additional file 1 for description of outcome measures). For the Turku center, Finnish translated versions of the SHI, MDADI, and EORTC-H&N35 were administered to study participants. The SHI, MDADI, and EORTC-H&N35 were completed at four time points: pre-operatively, and at 1 month, 6 month, and 1 year post-operative intervals as part of routine patient follow-up.

### Statistical analysis

This exploratory study investigated the impact of treatment modality and assessment time on patients’ self-perceived speech and swallowing function and quality of life. In addition to descriptive statistics, mixed effects regression models were fitted to every subscale of the three outcome measures: SHI, MDADI, and EORTC-H&N35, resulting in 18 models (see Additional files 2 and 3 for details of statistical analysis and participant recruitment flowchart, respectively). To account for the missing data, maximum likelihood estimates were used for estimating the parameters of the mixed effects linear regression models since they can handle missing data points robustly without discarding any data [25]. A Bonferroni correction was applied to the .05 alpha level to adjust for the 18 mixed effects regression analyses that were completed [26]. Hence, statistical significance was set a priori at the .0028 alpha level for final interpretation of mixed effects analyses. Statistical analyses were performed using SPSS (Version 22.0).

## Results

Table 1 displays patient demographic and treatment-related information. One hundred and seventeen patients participated in this study. Mean age of patients was 58.2 years with a standard deviation of 13.3 years. Males comprised 63% of study participants. Descriptive statistics for the SHI, MDADI, and EORTC-H&N35 are displayed in Additional file 4. Results of the mixed effects regression analyses identified differences in speech and swallowing function, and quality of life reported by patients with oral cancer of the tongue between pre- operation and follow-up assessment time points; treatment (by time) effects were also observed for the MDADI and EORTC-H&N35, but not for the SHI.

**Table 1:** Patient characteristics

Demographic	Variable	HNRN Site: No. (%)			
		Edmonton (*n* = 63)	New York (*n* = 41)	Turku (*n* = 13)	Total (*n* = 117)
Sex	Male	39 (62)	27 (66)	5 (38)	71 (61)
	Female	24 (38)	14 (34)	8 (62)	46 (39)
T-stage at diagnosis	T1	2 (3)	8 (20)	3 (23)	13 (11)
	T2	44 (70)	8 (20)	7 (54)	59 (50)
	T3	14 (22)	9 (22)	3 (23)	26 (22)
	T4	3 (5)	7 (17)	0 (0)	10 (9)
	Unknown	0 (0)	9 (22)	0 (0)	9 (8)
AJCC Staging	Stage	2 (3)	6 (15)	2 (15)	10 (9)
	Stage II	27 (43)	4 (10)	4 (31)	35 (29)
	Stage III	16 (25)	2 (5)	2 (15)	20 (17)
	Stage IVA	18 (29)	20 (49)	5 (38)	43 (37)
	Unknown	0 (0)	9 (22)	0 (0)	9 (8)
Treatment	Sx	29 (46)	15 (37)	6 (46)	50 (43)
	Sx/RT	17 (27)	10 (24)	0 (0)	27 (23)
	Sx/CRT	17 (27)	16 (39)	7 (54)	40 (34)
Surgical Approach	Transoral	54 (86)	21 (51)	8 (61)	83 (71)
	Transmandibular	6 (10)	12 (29)	1 (8)	19 (16)
	Unknown	3 (5)	8 (20)	4 (31)	15 (13)
Reconstruction	RFFF	40 (64)	19 (46)	7 (54)	66 (56)
	Other	7 (11)	6 (14)	0 (0)	13 (11)
	Unknown	16 (25)	16 (39)	6 (46)	38 (32)

*Abbreviations*: *AJCC* American Joint Committee on Cancer, *Sx* surgery, *Sx/RT* primary surgery and adjuvant radiation therapy, *Sx/CRT* primary surgery and adjuvant chemoradiation therapy, *RFFF* radial forearm free flap

### Speech function

Table 2 displays results of mixed effects regression models for the SHI outcome measure. Three mixed effects linear regression models were fitted to the SHI total and subscale (Psychosocial, Speech) scores. No statistically significant interactions between treatment and assessment time, nor any significant results for the main effect of treatment were found. At 1 month post-operation, statistically significantly higher total SHI (+10.0, *p* = .002) and Speech subscale (+5.4, *p* < .001) scores compared with baseline scores were observed for the combined cohort. At 6 months post-operation, a statistically significantly higher score was found for only the Speech subscale (+4.7, *p* = .002) of the SHI compared with baseline. At 1 year post-operation, no statistically significant results were found. No statistically significant results were found for the Psychosocial subscale of the SHI for any comparison period. However, according to the 6 point cut-off score established by the developers of the SHI [17], estimated mean total SHI scores at all time points (pre-op: 20.9, 1 month: 30.8, 6 months: 30.1, 1 year: 28.5) indicate clinically relevant self-reported speech impairment.

**Table 2:** Mixed effects linear regression models for Speech Handicap Index (SHI)

Subscale (n)	Variable	Estimate	95% CI	SE	*t*	*p*	SDs of Random Effects	
							Intercept	Residual
SHI (*n* = 83)	Baseline	20.86	15.6 to 26.2	2.71	7.69		16.86	16.14
	1 month	+9.98	+3.7 to +16.2	3.14	3.18	.002*		
	6 month	+9.24	+2.6 to +15.9	3.34	2.77	.006		
	1 year	+7.66	+.4 to +14.8	3.69	2.08	.040		
Psychosocial (*n* = 92)	Baseline	8.46	5.8 to 11.0	1.32	6.41		8.70	7.93
	1 month	+4.31	+1.2 to +7.4	1.55	2.79	.006		
	6 month	+4.12	+.9 to +7.3	1.65	2.50	.013		
	1 year	+4.04	+.5 to +7.6	1.80	2.25	.026		
Speech (*n* = 92)	Baseline	11.55	9.2 to 13.9	1.21	9.55		7.60	7.75
	1 month	+5.41	+2.7 to +8.1	1.36	3.96	<.001*		
	6 month	+4.65	+1.8 to +7.5	1.45	3.22	.002*		
	1 year	+3.16	0 to +6.3	1.58	2.00	.048		

Note.*p < .0028; Baseline = pre-operative estimate of mean subscale scores for combined cohort; 1 month/6 months/1 year = difference between combined cohort mean subscale scores and baseline mean for the respective assessment time point; *p*-value for baseline scores not provided given that it tests the hypothesis that the baseline mean is significantly different from 0, results of which are not meaningful; 95% CI calculated using bootstrapping

### Swallowing function

Table 3 displays results of mixed effects regression models for the MDADI outcome measure. Three mixed effects linear regression models were fitted to the MDADI subscale (Emotional, Functional, Physical) scores. Regression analyses for the MDADI subscale scores did not reveal any statistically significant interactions between treatment and assessment time. However, when the interaction term was removed from the models, treatment and time effects were revealed for all three MDADI subscales.

**Table 3:** Mixed effects linear regression models for M.D. Anderson Dysphagia Inventory

Subscale (n)	Variable	Estimate	95% CI	SE	*t*	*p*	SDs of Random Effects	
							Intercept	Residual
Emotional (*n* = 112)	Baseline	79.40	75.3 to 83.5	2.08	38.25		9.38	12.27
	Sx-RT	−4.58	−10.4 to +1.3	2.94	−1.56	.122		
	Sx-CRT	−8.64	−3.3 to −14.0	2.68	−3.22	.002*		
	1 month	−6.45	−2.7 to −10.2	1.90	−3.39	<.001*		
	6 month	−3.34	−7.2 to +.5	1.97	−1.69	.092		
	1 year	−3.61	−7.9 to +.6	2.16	−1.67	.095		
Functional (*n* = 112)	Baseline	84.32	79.6 to 89.0	2.38	35.48		11.70	12.93
	Sx-RT	−5.62	−12.3 to +1.2	3.45	−1.63	.107		
	Sx-CRT	−13.08	−6.8 to −19.3	3.15	−4.15	<.001*		
	1 month	−8.31	−4.4 to −12.3	2.02	−4.11	<.001*		
	6 month	−5.95	−1.9 to −10.1	2.09	−2.85	.005		
	1 year	−3.96	−8.5 to +.5	2.29	−1.73	.085		
Physical (*n* = 112)	Baseline	77.85	73.4 to 82.3	2.29	34.05		11.01	12.75
	Sx-RT	−7.34	−.8 to −13.8	3.30	−2.23	.028		
	Sx-CRT	−9.74	−3.9 to −15.5	3.01	−3.24	.002*		
	1 month	−7.09	−3.3 to −11.0	1.99	−3.57	<.001*		
	6 month	−3.58	−7.6 to +.5	2.06	−1.74	.084		
	1 year	−3.46	−8.0 to +.9	2.25	−1.54	.126		

*Note.* *p < .0028; Baseline = pre-operative mean estimate for surgery only group; Sx-RT,/Sx-CRT = difference between pre-operative mean scores and baseline for surgery and radiotherapy, and, surgery and chemoradiotherapy groups, respectively; 1 month/6 months/1 year = difference between mean subscale score and baseline for the respective assessment time point for combined cohort; *p*-value for baseline scores not provided given that it tests the hypothesis that the baseline mean is significantly different from 0, results of which are not clinically meaningful; 95% CI calculated using bootstrapping approach

### Treatment effects

Statistically significant greater impairment in the Emotional (−8.6, *p* = .002), Functional (−13.1, *p* < .001) and Physical (−9.7, *p* = .002) subscales of the MDADI were found for patients in the Sx-CRT group compared with baseline scores. No statistically or clinically relevant differences in MDADI subscale scores were found for patients in the Sx-RT group compared with baseline.

### Time effects

At 1 month post-operation, statistically significantly greater impairment was observed for the Emotional (−6.5, *p* < .001), Functional (−8.3, *p* < .001) and Physical (−7.1, *p* < .001) subscales of the MDADI compared with estimated mean baseline scores for the combined study cohort. No statistically significant differences were found between baseline and 6 months, and baseline and 1 year post-operation for the subscales of the MDADI. However, when examining individual scores, a clinically significant (≥ 20 point) drop in MDADI subscale scores between baseline and 1 month (33%, 33%, 23%), 6 months (22%, 30%, 31%), and 1 year (22%, 20%, 22%) for the Emotional, Functional and Physical subscales respectively, was observed in the present study.

### Quality of life related to head and neck cancer

Table 4 displays results of mixed effects regression models for the EORTC-H&N35 outcome measure. Twelve mixed effects linear regression models were fitted to the EORTC-H&N35 subscale (Pain, Swallowing, Senses, Speech, Eating, Social Contact, Sexuality, Teeth, Opening Mouth, Dry Mouth, Saliva, Cough) scores. Treatment by time interactions, treatment effects, and time effects were revealed for the EORTC-H&N35.

**Table 4:** Mixed effects linear regression models for EORTC Quality of Life Questionnaire Head and Neck Module (EORTC-H&N35)

Subscale (n)	Variable	Estimate	95% CI	SE	*t*	*p*	SDs of Random Effects	
							Intercept	Residual
Pain (*n* = 109)	Baseline	36.54	32.1 to 40.9	2.26	16.20		17.30	14.30
	1 month	−13.22	−8.7 to −17.8	2.32	−5.70	<.001*		
	6 month	−15.73	−11.0 to −21.0	2.40	−6.55	<.001*		
	1 year	−16.07	−11.0 to −20.4	2.55	−6.29	<.001*		
Swallowing (*n* = 109)	Baseline	11.63	6.1 to 17.2	2.86	4.07		12.58	16.70
	Sx-RT	+6.81	−1.0 to +14.7	4.00	1.70	.092		
	Sx-CRT	+10.36	+3.1 to +17.7	3.70	2.80	.006		
	1 month	+10.12	+4.9 to +15.4	2.66	3.80	<.001*		
	6 month	+5.46	0 to +10.8	2.76	1.98	.049		
	1 year	+1.06	−4.7 to +6.9	2.93	.36	.719		
Senses (*n* = 109)	Baseline	16.12	11.4 to 20.9	2.39	6.73		17.99	15.54
	1 month	+1.57	−3.4 to +6.6	2.51	.63	.533		
	6 month	+8.92	+3.8 to +14.2	2.60	3.43	<.001*		
	1 year	+2.24	−3.1 to +7.8	2.77	.81	.419		
Speech (*n* = 108)	Baseline	19.93	15.9 to 24.1	2.09	9.53		12.96	15.73
	1 month	+3.64	−1.4 to +8.6	2.51	1.45	.149		
	6 month	+2.78	−2.5 to +7.7	2.62	1.04	.302		
	1 year	−.68	−6.1 to +4.8	2.77	−.25	.806		
Eating (*n* = 108)	Baseline	20.50	13.2 to 27.8	3.69	5.55		15.78	21.84
	Sx-RT	+8.76	−1.2 to +18.8	5.13	1.71	.091		
	Sx-CRT	+14.25	+5.0 to 23.3	4.74	3.01	.003*		
	1 month	+2.19	−4.6 to +9.1	3.47	.63	.528		
	6 month	+3.12	−4.0 to +10.3	3.63	.86	.392		
	1 year	−2.95	−10.3 to +4.6	3.85	−.77	.445		
Social Contact (*n* = 108)	Baseline	10.47	6.7 to 14.4	1.96	5.33		13.09	14.06
	1 month	+4.26	−.2 to +8.8	2.26	1.89	.060		
	6 month	+3.97	−.6 to +8.5	2.35	1.69	.092		
	1 year	+3.94	−.8 to +8.8	2.49	1.59	.114		
Sexuality (*n* = 105)	Baseline	26.11	19.5 to 32.9	3.40	7.68		22.52	23.45
	1 month	+1.71	−6.1 to +9.5	3.93	.44	.664		
	6 month	+.51	−7.6 to +8.4	4.07	.13	.901		
	1 year	−7.38	−15.9 to +1.3	4.30	−1.71	.088		
Teeth (*n* = 109)	Baseline	23.38	17.2 to 29.6	3.20	7.30		16.92	26.13
	1 month	−3.39	−11.5 to +4.9	4.19	−.81	.419		
	6 month	−1.96	−10.3 to +6.5	4.30	−.46	.649		
	1 year	+.24	−8.8 to +9.3	4.64	.05	.958		
Open Mouth (*n* = 109)	Baseline	14.65	7.4 to 21.9	3.71	3.94		14.56	23.62
	Sx-RT	+2.24	−7.6 to +12.0	5.05	.44	.659		
	Sx-CRT	+14.59	+5.5 to +23.7	4.68	3.12	.002*		
	1 month	+12.42	+5.2 to +19.6	3.71	3.35	<.001*		
	6 month	+11.30	+3.7 to +18.9	3.88	2.92	.004		
	1 year	+2.86	−5.3 to +11.0	4.13	.69	.490		
Dry Mouth (*n* = 109)	Baseline	28.91	19.2 to 38.5	4.93	5.86		18.26	24.89
	Sx-RT	−15.39	−31.2 to +.6	8.05	−1.91	.057		
	Sx-CRT	−6.05	−20.4 to +8.4	7.32	−.83	.409		
	1 m x Sx	+8.23	−4.0 to +20.1	6.13	1.34	.180		
	6 m x Sx	+5.08	−7.4 to +17.5	6.39	.80	.428		
	1y x Sx	+6.16	−7.1 to +20.0	6.87	.90	.371		
	1 m x Sx-RT	+3.83	−15.5 to +23.7	10.11	.38	.705		
	6 m x Sx-RT	+42.18	+22.5 to +62.9	10.18	4.14	<.001*		
	1y x Sx-RT	+35.27	+13.7 to +56.9	10.96	3.22	.001*		
	1 m x Sx-CRT	+14.88	−3.0 to +32.5	9.12	1.63	.104		
	6 m x Sx-CRT	+36.45	+17.8 to +55.0	9.63	3.79	<.001*		
	1y x Sx-CRT	+33.03	+13.0 to +52.6	10.18	3.25	.001*		
Sticky Saliva (*n* = 109)	Baseline	25.74	19.3 to 32.3	3.34	7.72		18.80	26.18
	1 month	+7.69	−.7 to +15.8	4.17	1.85	.066		
	6 month	+15.30	+6.7 to +23.9	4.35	3.53	<.001*		
	1 year	+15.34	+6.2 to +24.5	4.63	3.33	.001*		
Cough (*n* = 109)	Baseline	17.74	13.4 to 22.0	2.20	8.05		12.20	17.56
	1 month	+3.82	−1.4 to +9.2	2.78	1.38	.170		
	6 month	+6.28	+.5 to +12.0	2.90	2.16	.032		
	1 year	−3.08	−9.2 to +3.00	3.08	−1.00	.318		

*Note.* *p < .0028; Models with treatment, time, and treatment x time interaction: Baseline = pre-operative mean estimate for surgery only group; Sx-RT/Sx-CRT = difference between pre-operative mean scores and baseline for surgery and radiotherapy, and, surgery and chemoradiotherapy groups, respectively; 1 m/6 m/1y x Sx/Sx-RT/Sx-CRT = difference between mean subscale scores and baseline at 1 month, 6 months, and 1 year post-operation, respectively, for the surgery only, surgery and radiotherapy, and, surgery and chemoradiotherapy groups, respectively; Models with treatment and time (no interaction term): Baseline = pre-operative mean estimate for surgery only group; Sx-RT,/Sx-CRT = difference between pre-operative mean scores and baseline for surgery and radiotherapy, and, surgery and chemoradiotherapy groups, respectively; 1 month/6 months/1 year = difference between mean subscale scores and baseline for respective assessment time point for combined cohort; Models with time variable only: Baseline = pre-operative mean estimate for subscale scores for combined cohort; 1 month/6 months/1 year = difference between mean subscale scores and baseline for respective assessment time point for combined cohort; *p*-value for baseline scores not provided given that it tests the hypothesis that the baseline mean is significantly different from 0, results of which are not clinically meaningful; 95% CI calculated using bootstrapping approach

### Treatment by time interactions

Statistically significant treatment by time interactions were observed for only the Dry Mouth subscale of the EORTC-H&N35. At 1 month post-operation, clinically (>10 points) significant worse Dry Mouth scores were observed for the Sx-CRT group (+14.9, *p* = .104) compared with baseline. At 6 months post-operation, clinically and statistically worse symptoms of Dry Mouth were observed for both the Sx-RT (+42.2, *p* < .001), and Sx-CRT (+36.5, *p* < .001) groups compared with baseline. At 1 year post-operation, Dry Mouth scores remained clinically and statistically worse than baseline for both the Sx-RT (+35.3, *p* < .001) and Sx-CRT (+33.03, *p* < .001) groups. No clinically or statistically significant differences in Dry Mouth were found between baseline and all post-operative assessments for the Sx group.

### Treatment effects

For three outcome variables of the EORTC-H&N35 (Eating, Opening Mouth, Swallowing), an independent treatment effect was observed. Statistically and clinically significantly greater difficulties with Eating (+14.3, *p* = .003), and Opening Mouth (+14.6, *p* = .002), and clinically (>10 points) greater difficulties with Swallowing (+10.4, *p* = .006), were reported for the Sx-CRT group compared with baseline. No significant differences were observed for the Sx-RT group compared with baseline for Eating, Opening Mouth, or Swallowing.

### Time effects

For five outcome variables of the EORTC-H&N35 (Pain, Swallowing, Senses, Open Mouth, Sticky Saliva), mixed effects regression models revealed differences in estimated mean scores between baseline and post-operative assessment scores for the combined cohort. At 1 month post-operation, statistically and clinically significantly less Pain (−13.2, *p* < .001), and more difficulties with Swallowing (+10.1, *p* < .001), and Opening Mouth (+12.4, *p* < .001) compared with baseline were observed. At 6 months post-operation, statistically and clinically significantly less Pain (−15.7, *p* < .001) and more problems with Sticky Saliva (+15.3, *p* < .001), clinically (>10 points) more difficulties with Opening Mouth (+11.3, *p* = .004), and statistically (but not clinically) significantly greater impairments with Senses (+8.9, *p* < .001) compared to baseline were found. No statistically or clinically significant treatment by time interactions, treatment effects, or time effects were found for Speech, Social Contact, Sexuality, Teeth, or Cough subscales of the EORTC-H&N35.

## Discussion

The purpose of this exploratory study was to evaluate self-reported speech and swallowing function, as well as quality of life, over the course of the first year post-operation and the impact of treatment modality on functional outcome in patients surgically treated for tongue cancer. To our knowledge, this is the first longitudinal study evaluating the functional and quality of life effects of surgical treatment for SCCA in patients suffering with cancer of the oral tongue. This multi-center study of the HNRN included a large sample (*n* = 117) of patients with cancer of the anterior tongue. By allowing institutions to collaborate in a structured manner, the HNRN approach offers a valuable research method for advancing functional outcomes research in HNC. Results indicated no significant differences in swallowing function by 6 months post-surgery and no significant differences in speech function by 1 year post-surgery compared with baseline. Decrements in multiple domains regarding quality of life were reported at all three post-operative follow-up assessments relative to baseline scores. Furthermore, treatment (by time) effects were revealed for swallowing and quality of life outcomes but not for speech outcomes.

### Speech function

Patients reported statistically significantly worse SHI scores at 1- and 6-months post-operation compared with pre-operative scores. These findings indicate that by the 1 year follow-up assessment, speech function was no longer statistically different from baseline levels. However, estimated mean total SHI scores for the combined cohort indicated clinically relevant (> 6 points) speech impairment at all four assessment time points (mean estimates 20.9 to 30.8). Previous investigations indicate that patients treated for oral cancer experience long-term speech problems [12, 17, 18]. For example, in a cross-sectional study of patients with head and neck cancer that were an average of 78 months out of surgery (with or without C/RT), Dwivedi et al. [12] reported a mean total SHI score of 34.4 for the oral cavity cancer subgroup, suggesting speech impairment may be present many years following surgical intervention.

No differences in Psychosocial scores were revealed for any comparison period in the present study, suggesting that psychosocial aspects of speech function were not significantly impaired post-operatively relative to baseline. Similarly, in a cross-sectional study of oral and oropharyngeal patients between 6 and 155 months out of surgery (with or without RT), patients reported more functional than psychosocial problems [27]. Treatment, and treatment by time effects were not revealed for any of the subscales of the SHI in the present study, suggesting that treatment modality does not differentially influence the functional or psychosocial aspects of speech in patients surgically treated for oral cancer. Previous studies utilizing the SHI evaluated combined groups of patients with oral and oropharyngeal cancer [12, 17, 18, 27], and more diverse head and neck cancer subsites [28, 29], and were often cross-sectional in nature [12, 17, 18, 27, 28], limiting comparisons of study findings.

### Swallowing function

Patient-reported swallowing outcomes revealed more difficulties with swallowing at 1 month post-operation compared with baseline. These swallowing difficulties affected patients’ emotional states, physical and functional abilities. By 6 months post-operation, this difference was no longer apparent. Thus, only short-term declines in swallowing function were found for the present study cohort. However, when examining individual scores, clinically relevant impairments in swallowing function (≥ 20-point drop in MDADI subscale scores) were observed for approximately 20% of participants in the present study at 1 year post-operation compared with baseline. As such, while a return to baseline MDADI subscale scores was observed by 6 months post-operation for the study cohort, large individual differences in swallowing function were found.

To the authors’ knowledge, no previous studies have longitudinally evaluated swallowing-related quality of life in patients surgically treated for tongue cancer using the MDADI. In a cross-sectional study of patients treated with primary surgery (+/− C/RT) who were on average 76 months post-treatment, mean MDADI scores for the oral cavity group were 71.7, 77.5, and 70.8 for the Emotional, Functional, and Physical subscales of the MDADI respectively [30]; these scores are similar to those observed in the present study. Another study found near-normal swallowing function for 26 patients treated for oral cavity SCCA assessed an average 15 years out of treatment [31]. Results of the present study and literature suggest that short-term decrements in swallowing function occur post-surgery in patients with oral SCCA, after which point patients start to recover their swallowing function, with patients reporting near-normal swallowing function many years post-treatment.

In the present study, patient-reported swallowing deficits were reported as worse by the Sx-CRT group compared to the Sx-RT group. These findings suggest that the addition of multiple adjuvant treatment interventions post-operation (i.e. chemo and RT) may negatively impact swallowing function. However, results of the present study may be confounded by differential disease progression before treatment. Published works on treatment influences on MDADI subscales are inconclusive. Shin et al. [6] reported significantly better swallowing capacity for glossectomy patients treated with surgery only compared with Sx-RT patients [6]. In contrast, Dwivedi et al. [30] found no statistically significant differences in mean MDADI subscales scores between the Sx, Sx-RT, and Sx-CRT groups of patients with oral or oropharyngeal cancer. Future work to discern if adjuvant C/RT affects post-operative patient-reported swallowing function in individuals with oral cancer is warranted.

### Quality of life related to head and neck cancer

Clinically (>10 point) and statistically significant treatment by time interactions were revealed for only the Dry Mouth subscale of the EORTC-H&N35, with problems starting after the onset of radiation therapy. Dry mouth continued to be a significant problem at 1 year post-operation for both groups. No clinically or statistically significant differences in dry mouth were found between baseline and all post-operative assessments for the Sx only group. Nordgren et al. [32] also found that patients with oral carcinoma who were treated with surgery only did not have problems with dry mouth, while persistent severe problems with dry mouth over time (3 months, 1 year and 5 years post-treatment) were reported by patients in the RT and combined treatment groups [32]. Chronic dry mouth is known to be a common side effect of treatment, particularly as a result of radiation therapy for oral cancer treatment and is documented well in the literature [5, 9, 32–35].

In addition to the dry mouth subscale, negative treatment effects of Sx-CRT (but not Sx-RT) were revealed for swallowing, eating, and opening mouth; these results suggest that patients treated with multiple adjuvant regimens may be impacted more negatively than patients treated with adjuvant RT only. These differences may also reflect an underlying time dependent trend that was not strong enough to reach statistical significance. Alternatively, these differences may not be caused by treatment but may reflect pre-operative differences between treatment groups.

Decreased pain at all follow-up appointments compared with pre-treatment scores were found, indicating a positive result in symptomatology for patients undergoing surgical removal of an anterior tongue carcinoma. However, there were long term difficulties with sticky saliva (6 months and 1 year post-operation). Given the time horizon, the greater difficulties with sticky saliva that were found at 6 and 12 months post-operation compared with baseline for the combined cohort likely reflect the added negative effect of radiation, while short-term impairments in senses (taste and smell) and opening mouth may be a result of both surgical and/or radiation induced impairments.

Similar difficulties in multiple domains of quality of life in patients with oral cancer have been reported in the literature [5, 29, 36, 37]. Petruson et al. [5] conducted a longitudinal study of 90 patients with oral or oropharyngeal cancer treated with combinations of brachytherapy, surgery, and C/RT. Similar to findings of the present study, Petruson et al. [5] found significantly more difficulties with dry mouth at all post-treatment time points (i.e., 3-, 12-, and 36-months compared with baseline) and short-term impairment in swallowing function (3 months post-treatment compared with baseline). In contrast to our study findings, Petruson et al. [5] also found short-term problems with pain (3 months versus baseline), and clinically significantly (> 10 points) more difficulty with teeth (3 months and 1 year post-treatment versus baseline) [5]. Lazarus et al. [29] examined EORTC-H&N35 scores from pre-treatment to 3 and 6 months post-treatment in patients with head and neck cancer treated with chemoradiotherapy. Results indicated continued difficulty with sticky saliva, speech, senses and dry mouth, at 6 months post-treatment [29]. Finally, in a longitudinal study of 80 patients with advanced oral or oropharyngeal cancer treated with reconstructive surgery and adjuvant radiotherapy at 6 months, patients reported less pain, and more problems swallowing, senses, social contact, teeth, opening mouth, dry mouth, sticky saliva, and coughing compared with baseline; difficulties with dry mouth, senses, opening mouth, sticky saliva, and coughing continued to be present at 1 year post-treatment [36]. Results of the present study of patients with oral cavity cancer and previous literature studying oral, oropharyngeal, and other head and neck subsites, found a consistent trend of long-term difficulties with dry mouth and sticky saliva. Differences in study findings on other EORTC-H&N35 subscales may be due to differences in treatment modalities, tumor sites and tumour stages of study samples.

## Conclusions

Results of the present study indicate that patients with cancer of the oral tongue who undergo surgical resection and reconstruction with or without adjuvant C/RT experience impairments in function and quality of life. Although SHI speech scores returned to baseline levels by the 1 year post-operation assessment, speech was clinically impaired at all assessment time points (baseline to 1 year post-operation). Short-term declines in swallowing function (MDADI and EORTC-H&N subscales) and most quality of life domains related to the head and neck returned to baseline levels by 1 year post-operation, while difficulties with dry mouth and sticky saliva persisted. A clear time trend of adjuvant (chemo)radiation therapy negatively affecting dry mouth scores over time was identified in this study, while negative independent effects of Sx-CRT on MDADI swallowing, and EORTC-H&N35 swallowing, eating, and opening mouth subscales were found.

Results of the present investigation will help guide clinical care and will be useful for patient counseling on expected short and long-term speech, swallowing, and quality of life outcomes of surgical and adjuvant treatment for oral cavity cancer. An increased utilization of patient-reported outcome measures in the literature reflects a move towards such patient-centred care.

Limitations of the present study include a large amount of missing responses (39%), clinical differences (i.e., T-stage and AJCC stage) found between responders and nonresponders at post-operative assessments, and the exclusion of other clinical and demographic variables such as sex, age, study site and TNM stage, in the mixed effects models due to risk of model overfit. Results of the present study suggest that treatment modality may differentially affect swallowing and quality of life outcomes in patients with oral cancer, while other studies also reported the influence of treatment modality [32], and other demographic and clinical factors (TNM stage, sex, age, tumour site) on speech [17], swallowing [6, 12, 38], and quality of life [32]. Future work to delineate how clinical and demographic factors differentially influence speech, swallowing and quality of life over time in patients treated for oral cavity cancer is warranted.

## Additional files

Additional file 1: Description of Outcome Measures. A review of study outcome measures and their psychometric properties. (DOCX 17 kb)

Additional file 2: Details of Statistical Analysis. Details of the mixed effects regression models conducted in this study and evaluation of missing data. (DOCX 15 kb)

Additional file 3: Figure S1. Flowchart of participant recruitment and retention rates for each HNRN site (i.e., Edmonton, New York, Turku). (DOCX 54 kb)

Additional file 4: Baseline and post-operative descriptive statistics for outcome measures. Samples sizes and measures of central tendency for subscales of the SHI, MDADI and EORTC-H&N35. (DOCX 16 kb)

## Abbreviations

AJCC: American Joint Committee on Cancer
CRT: Chemoradiation therapy
EORTC-H&N35: European Organization for Research and Treatment of Cancer Quality of Life Questionnaire Head and Neck Module
HNC: Head and neck cancer
HNRN: Head and Neck Research Network
MDADI: M.D. Anderson Dysphagia Inventory
RT: Radiation therapy
SCCA: Squamous cell carcinoma
SHI: Speech Handicap Index
Sx: Primary surgery only
Sx-CRT: Primary surgery and adjuvant chemoradiation therapy
Sx-RT: Primary surgery and adjuvant radiation therapy
