# Correction to: Functional and quality of life outcomes after partial glossectomy: a multi-institutional longitudinal study of the head and neck research network

**DOI:** 10.1186/s40463-017-0236-9

**Published:** 2017-10-05

**Authors:** Agnieszka Dzioba, Daniel Aalto, Georgina Papadopoulos-Nydam, Hadi Seikaly, Jana Rieger, Johan Wolfaardt, Martin Osswald, Jeffrey R. Harris, Daniel A. O’Connell, Cathy Lazarus, Mark Urken, Ilya Likhterov, Raymond L. Chai, Erika Rauscher, Daniel Buchbinder, Devin Okay, Risto-Pekka Happonen, Ilpo Kinnunen, Heikki Irjala, Tero Soukka, Juhani Laine, Head and Neck Research Network

**Affiliations:** 1 Department of Surgery, University of Alberta Hospital, Edmonton, AB T6G 2G4 Canada; 2 Institute for Reconstructive Sciences in Medicine (iRSM), Misericordia Community Hospital, Edmonton, AB Canada; 3 Rehabilitation Medicine, Communication Sciences and Disorders, University of Alberta, Edmonton, AB Canada; 4 Division of Otolaryngology-Head and Neck Surgery, University of Alberta, Edmonton, AB Canada; 5 Division of Head and Neck Surgery, Department of Otolaryngology, Head & Neck Surgery, Mount Sinai Beth Israel, New York, NY USA; 6 Thyroid, Head And Neck Cancer (THANC) Foundation, New York, NY USA; 7 Department of Oral and Maxillofacial Surgery, Mount Sinai Beth Israel, New York, USA; 8 Department of Oral and Maxillofacial Diseases, Turku University Hospital, Turku, Finland; 9 Department of Oral and Maxillofacial Surgery, University of Turku, Turku, Finland; 10 Department of Oto-Rhino-Laryngology, Turku University Hospital, Turku, Finland

## Erratum to: Journal of otolaryngology (2017) 46:56 DOI: 10.1186/s40463-017-0234-y

In the original version of this article [1], published on 4 September 2017, the name of author ‘Heikki Irjala’ was wrongly displayed.

Originally the author name has been published as:

- Irjala Heikki

The correct author name is:

- Heikki Irjala

The original publication of this article has been corrected.
