# Supplementary material for: Functional and quality of life outcomes after partial glossectomy: a multi-institutional longitudinal study of the head and neck research network
Source: J Otolaryngol Head Neck Surg. 2017 Sep 4;46:56. doi: 10.1186/s40463-017-0234-y (PMC5583999; doi:10.1186/s40463-017-0234-y)
Supplement: Supplementary file 1 — Description of Outcome Measures. A review of study outcome measures and their psychometric properties. (DOCX 17 kb) [file 40463_2017_234_MOESM1_ESM.docx]

**Description of Outcome Measures**

The SHI assesses patient-reported difficulties related to speaking function. This measure consists of 30 items utilizing a five-point Likert scale (0 = Never; 4 = Always). A total SHI score is derived by summing all individual responses and ranges from 0 to 120 [1, 2]. Two subscale scores are also generated: the Speech Function (SF) score, and the Psychosocial Functioning (PF) score, each consisting of 14 items, with total subscale scores ranging from 0 to 56 [3]. Higher scores indicate greater speech-related problems. Based on sensitivity and specificity analyses, a total SHI score of 6 or higher identifies patients with speech problems in daily life [2]. Good levels of validity and reliability of the SHI have been reported [2]. Cronbach’s alpha for the SHI in the present study was .93.

The MDADI was developed to evaluate the self-perceived swallowing function of individuals and consists of 20 items utilizing a 5-point Likert scale (Strongly Agree to Strongly Disagree) [4]. The MDADI has three subscales: Emotional, Functional, and Physical, which address how an individual’s swallowing disorder affects their emotional wellbeing, daily activities, and self-perceived physical difficulties with swallowing. Each subscale score is summed, and transformed to a score with a range of 0 (extremely low functioning) to 100 (high functioning) [4]. A change of 20 points in individual MDADI subscale scores over time has been suggested to be clinically meaningful as it reflects a consistent shift of responses moving up or down one level on the five point Likert scale [5, 6]. Good levels of validity and reliability of the MDADI have been reported [4]. Cronbach’s alpha for the MDADI in the present study was .92.

The EORTC-H&N35 questionnaire was developed to evaluate quality of life issues in a wide range of head and neck cancer patients [7]. The instrument consists of 35 items comprising 7 subscales: pain, swallowing, senses, speech, social eating, social contact, and sexuality. In addition, there are single items covering problems with teeth, dry mouth, sticky saliva, cough, and opening the mouth wide. Furthermore, there are single items with dichotomous response scales covering weight loss, weight gain, nutritional supplements, feeding tubes, and pain medication, which were not included in analyses in the present investigation. EORTC-H&N35 scales and items were scored and linearly transformed to scales of 0 to 100, with higher scores indicating greater impairment [8]. A 10 point or higher difference in subscale scores indicates clinically relevant changes [9]. Good validity and reliability scores for the EORTC-H&N35 have been observed [7, 8]. Cronbach’s alpha for the EORTC-H&N35 in the present study was .94.

**References**

1. Dwivedi RC, St. Rose S, Chisholm EJ, Bisase B, Amen F, Nutting CM, et al. Evaluation of speech outcomes using English version of the Speech Handicap Index in a cohort of head and neck cancer patients. Oral Oncol. 2012;48(6):547-553.

2. Rinkel RN, Verdonck-de Leeuw IM, van Reij EJ, Aaronson NK, Leemans CR. Speech Handicap Index in patients with oral and pharyngeal cancer: Better understanding of patients' complaints. Head Neck. 2008;30(7):868-874.

3. Rinkel, RN, Verdonck-de Leeuwe IM, de Bree R, Aaronson NK, Leemans CR. Validity of patient-reported swallowing and speech outcomes in relation to objectively measured oral function among patients treated for oral or oropharyngeal cancer. Dysphagia. 2015;30(2):196-204.

4. Chen AY, Frankowski R, Bishop-Leone J, Hebert T, Leyk S, Lewin J, et al. The development and validation of a dysphagia-specific quality-of-life questionnaire for patients with head and neck cancer: The M. D. Anderson Dysphagia Inventory. Arch Otolaryngol Head Neck Surg. 2001;127(7):870-876.

5. Hutcheson KA, Barrow MP, Lisec A, Barringer DA, Gries K, Lewin JS. What is a clinically relevant difference in MDADI scores between groups of head and neck cancer patients? Laryngoscope. 2016;126(5):1108-1113.

6. Lu W, Wayne PM, Davis RB, Buring JE, Li H, Goguen LA, et al. Acupuncture for dysphagia after chemoradiation in head and neck cancer: Rationale and design of a randomized, sham-controlled trial. Contemp Clin Trials. 2012;33(4):700-711.

7. Bjordal K, Hammerlid E, Ahlner-Elmqvist M, de Graeff A, Boysen M, Evensen JF, et al. Quality of life in head and neck cancer patients: Validation of the European Organization for Research and Treatment of Cancer Quality of Life Questionnaire-H&N35. J Clin Oncol. 1999;17(3):1008-1019.

8. Bjordal K, de Graeff A, Fayers PM, Hammerlid E, van Pottelsberghe C, Curran D, et al. A 12 country field study of the EORTC QLQ-C30 (version 3.0) and the head and neck cancer specific module (EORTC QLQ-H&N35) in head and neck patients. Euro J Cancer. 2000;36(14):1796-1807.

9. Passchier E, Stuiver M, van der Molen L, Kerkhof S, van den Brekel M, Hilgers F. Feasibility and impact of a dedicated multidisciplinary rehabilitation program on health-related quality of life in advanced head and neck cancer patients. Eur Arch Otorhinolaryngol. 2016;273(6):1577-1587.
