# Supplementary material for: Functional and quality of life outcomes after partial glossectomy: a multi-institutional longitudinal study of the head and neck research network
Source: J Otolaryngol Head Neck Surg. 2017 Sep 4;46:56. doi: 10.1186/s40463-017-0234-y (PMC5583999; doi:10.1186/s40463-017-0234-y)
Supplement: Supplementary file 2 — Details of Statistical Analysis. Details of the mixed effects regression models conducted in this study and evaluation of missing data. (DOCX 15 kb) [file 40463_2017_234_MOESM2_ESM.docx]

Details of Statistical Analysis

The eighteen subscale scores were input separately, each as a dependent variable; treatment modality (three levels: Sx, Sx-RT and Sx-CRT) and assessment time (four levels: pre-op, and 1-, 6-, and 12-months post-op) and their interactions, were input as independent variables; a random intercept for patients was included in each model to account for variability in individual response patterns.  A two step process for reducing the mixed models was undertaken: 1) If the interaction terms were statistically non-significant (p >.05), a model excluding interactions was reported to evaluate the independent effects of treatment and assessment time on subscale scores; 2). If the treatment variable was statistically non-significant, treatment was further dropped from the model, and only a model including time as the independent variable was reported.

The authors considered including other clinical and demographic variables (i.e., T stage, AJCC stage, sex, Research site) in the regression models. However, including these variables in the models would have increased the number of statistical parameters considerably risking over interpretation of data. In addition, when interpreting the results, the impact of treatment may be confounded by differences in the pathologies. While treatment variables naturally depended on AJCC stage (**χ^2^** = 25.2, *df* = 6, *p* <.001), treatment modality was statistically independent across sites (**χ^2^** = 6.7, *df* = 4, *p* =.15).

In the present data, 39% of the responses were missing. Several reasons for incomplete data were observed in the present study including patients choosing not to respond to questionnaire items deemed too sensitive (i.e., some patients chose not to respond to the sexuality subscale of the EORTC-H&N35), missed appointments, traveling difficulties to post-operative functional assessment clinics, health issues and death. Twenty patients died during the 1 year follow-up assessment (one before the 1 month assessment, seven before the 6 month assessment, and twelve before the 1 year assessment) and six patients experienced a disease recurrence during the study follow-up (three before the 6 month assessment and three before the 1 year assessment). A logistic regression model was used to analyze the pattern of missing values. The strongest predictors for missing values were time (missing values at pre-op: 23%; 1 month: 35%; 6 months: 43%; 1 year: 54%), treatment (missing values for Sx: 43%; Sx-RT: 29%; Sx-CRT: 41%), and site (Edmonton: 31%; New York: 55%; Turku: 27%). Participant recruitment and retention rates segregated by research site are displayed in in Additional file 3. No statistically significant differences between responders and non-responders were identified for sex, T-stage, AJCC stage or treatment at baseline and 1 month post-op, while statistically significant differences between responders and non-responders were found at 6 months post-op and 1 year post-op for T-stage (6 months: **χ^2^** = 11.30, *df* = 3, *p* = .01, 1 year: **χ^2^** = 13.16, *df* = 3, *p* = .004), and AJCC stage (6 months: **χ^2^** = 11.94, *df* = 3, *p* = .008; 1 year: **χ^2^** = 13.67, *df* = 3, *p* = .003).
