# Supplementary material for: Functional and quality of life outcomes after partial glossectomy: a multi-institutional longitudinal study of the head and neck research network
Source: J Otolaryngol Head Neck Surg. 2017 Sep 4;46:56. doi: 10.1186/s40463-017-0234-y (PMC5583999; doi:10.1186/s40463-017-0234-y)
Supplement: Supplementary file 3 — Flowchart of participant recruitment and retention rates for each HNRN site (i.e., Edmonton, New York, Turku). (DOCX 54 kb) [file 40463_2017_234_MOESM3_ESM.docx]

## Edmonton

## Turku

## New York

Assessed for eligibility (N/A)

Excluded (N/A)

**Included (n=13)**

Assessed for eligibility (N/A)

Excluded (N/A)

**Included (n=41)**

Assessed for eligibility (n= 77)

Excluded (n=14)

- Not meeting inclusion criteria (n=8)
- Declined to participate (n = 6)

**Included (n=63)**

Pre-treatment

n=13 (100%)

Pre-treatment

n=32 (78%)

Pre-treatment

n=52 (83%)

­

1-month

n=30 (73%)

1-month

n=11 (85%)

1-month

n=45 (71%)

­­­­­­

6-months

n=11 (85%)

6-months

n=16 (39%)

6-months

n=47 (75%)

1-year

n=8 (20%)

1-year

n=11 (85%)

1-year

n=39 (62%)

***Figure 1.***
