# Supplementary material for: Functional and quality of life outcomes after partial glossectomy: a multi-institutional longitudinal study of the head and neck research network
Source: J Otolaryngol Head Neck Surg. 2017 Sep 4;46:56. doi: 10.1186/s40463-017-0234-y (PMC5583999; doi:10.1186/s40463-017-0234-y)
Supplement: Supplementary file 4 — Baseline and post-operative descriptive statistics for outcome measures. Samples sizes and measures of central tendency for subscales of the SHI, MDADI and EORTC-H&N35. (DOCX 16 kb) [file 40463_2017_234_MOESM4_ESM.docx]

| Baseline and post-operative descriptive statistics for outcome measures | | | | | | | | | | | | | |
| --- | --- | --- | --- | --- | --- | --- | --- | --- | --- | --- | --- | --- | --- |
| Scale | | Baseline  *N* Mean (SD) *M* (IQR) | | | 1 month post-op  *N*  Mean (SD) *M* (IQR) | | | 6 months post-op  *N* Mean (SD) *M* (IQR) | | | 1 year post-op  *N* Mean (SD) *M* (IQR) | | |
| SHI  SHI Score  Psychosocial  Speech  MDADI  Emotional  Functional  Physical  EORTC Pain  Swallow  Senses  Speech  Eating  Contact  Sexuality  Teeth  Open Mouth  Dry Mouth  Saliva  Cough | 71  76  76  97  97  97  93  92  93  92  92  92  87  93  93  93  91  92 | | 20.08 (22.83)  8.36 (11.01)  11.18 (11.53)  75.43 (15.32)  78.49 (10.05)  73.18 (19.66)  36.93 (24.95)  16.27 (19.81)  15.61 (25.76)  19.53 (22.19)  27.34 (29.24)  9.85 (17.24)  25.28 (32.53)  23.26 (31.02)  19.67 (28.76)  22.17 (29.23)  24.50 (29.76)  17.31 (21.77) | 10 (33)  2 (16)  8 (17)  73 (20)  80 (28)  75 (36)  33 (33)  8 (31)  0 (17)  11 (33)  17 (40)  0 (13)  17 (33)  0 (33)  0 (33)  0 (33)  0 (33)  0 (33) | 58  63  63  83  83  83  79  79  80  79  79  79  73  75  80  79  80  80 | 29.48 (19.65)  12.44 (10.50)  16.25 (9.11)  69.20 (15.56)  70.42 (17.49)  66.29 (14.41)  20.90 (18.97)  25.34 (19.08)  15.87 (19.64)  22.80 (17.26)  29.17 (23.46)  14.60 (18.49)  27.12 (34.17)  18.19 (28.63)  32.03 (26.82)  37.07 (33.37)  31.62 (29.53)  20.72 (20.07) | 29.5 (33)  13 (18)  17 (15)  72 (23)  72 (24)  65 (17)  17 (25)  25 (25)  17 (29)  22 (22)  25 (34)  7 (27)  0 (50)  0 (33)  33 (58)  33 (67)  33 (33)  33 (33) | 48  53  53  73  73  73  70  70  70  69  68  69  65  69  69  70  69  69 | 29.44 (23.51)  12.43 (11.90)  15.70 (10.47)  72.96 (16.30)  73.86 (19.08)  70.95 (16.20)  18.46 (17.87)  20.81 (24.03)  24.63 (25.82)  21.89 (20.14)  28.57 (28.21)  13.81 (20.43)  26.64 (33.30)  21.68 (31.22)  28.90 (30.21)  49.98 (32.56)  39.09 (32.38)  23.57 (22.93) | 25.5 (38)  9 (23)  17 (15)  77 (22)  80 (30)  72 (20)  16.8 (25)  8 (33)  17 (33)  22 (33)  17 (42)  7 (20)  17 (33)  0 (33)  33 (33)  50 (34)  33 (67)  33 (33) | 36  42  42  56  56  56  57  57  57  57  56  57  53  54  56  57  56  57 | 26.97 (28.17)  11.74 (14.48)  13.74 (11.94)  73.66 (16.02)  76.98 (16.77)  71.89 (17.62)  18.61 (22.88)  16.45 (21.42)  17.50 (21.42)  18.05 (19.64)  22.03 (27.65)  12.92 (20.16)  17.30 (27.93)  24.03 (33.91)  20.78 (26.64)  47.92 (32.82)  40.44 (37.49)  14.55 (20.85) | 14 (46)  5 (21)  10.5 (19)  73 (26)  80 (23)  70 (27)  17 (25)  8 (25)  17 (33)  11 (28)  8 (33)  0 (20)  0 (33)  0 (33)  0 (33)  33 (34)  33 (67)  0 (33) |

*Note. SD =* standard deviation, *M* = Median, IQR = Interquartile range
